# Exploring factors improving support for vaccinations among Polish primary care physicians

**DOI:** 10.1371/journal.pone.0232722

**Published:** 2020-05-01

**Authors:** Pawel Stefanoff, Tomasz Sobierajski, Helena Bulinska-Stangrecka, Ewa Augustynowicz

**Affiliations:** 1 Department of Epidemiology of Infectious Diseases and Surveillance, National Institute of Public Health – National Institute of Hygiene, Warsaw, Poland; 2 Division of Infection Control and Environmental Health, Norwegian Institute of Public Health, Oslo, Norway; 3 Institute of Applied Social Sciences, University of Warsaw, Warsaw, Poland; 4 Faculty of Administration and Social Sciences, Warsaw University of Technology, Warsaw, Poland; University of Campania, ITALY

## Abstract

In Poland, primary care physicians are the most used and most trusted source of information on immunisation. We aimed to explore factors influencing support for vaccinations among physicians employed in the childhood immunisation programme, in order to inform education of healthcare workers and programme organization. In June-July 2017, we carried out a national cross-sectional survey of physicians working in randomly selected primary healthcare practices, and interviewed them by telephone. We assessed support for vaccinations using an ordinal scale (0–6) comprised of three equally weighted questions on the respondent support of the programme and vaccination of self and family. We also created a scale (0–3) based on correct answers to vaccination myths. We used ordered logistic regression to investigate factors independently influencing support for vaccinations, reporting the proportional odds ratios and 95% confidence intervals for one unit increase in the support score. Of 2,609 respondents contacted, we interviewed 500 (19%). The median vaccination support score (0–6) was 5 (IQR 2). After adjusting for other variables, we did not find significant effects of sex, medical specialty, adhering to recommendations, attending a conference in previous year, using non-scientific sources of information and self-assessed knowledge on vaccination support score. Age over 60 years, correctly addressing vaccination myths and use of one or more than one scientific sources of knowledge, significantly improved support for vaccinations (aOR = 1.97, 1.57, 3.09 and 2.68, respectively). We recommend to increase the amount, quality and accessibility of evidence-based educational materials for primary care physicians working with childhood immunisations.

## Introduction

Confidence in vaccination programmes is crucial for maintaining high vaccine uptake [1, 2]. A systematic review of 68 investigations indicated that childhood vaccination coverage was determined by parents’ perception of vaccines as safe, a positive recommendation by the general practitioner, fewer practical barriers to vaccinate and general positive attitude towards vaccination [3]. Vaccine acceptance or refusal are driven to a large degree by vaccine confidence, defined as the trust in the effectiveness and safety of vaccines [1, 2]. Therefore, well informed healthcare professionals can be a reliable and trusted source of information for parents and influence their decision to vaccinate their children [4–9]. In 2015 and 2018, large-scale surveys of vaccine confidence in all 28 EU member states, commissioned by the European Commission, showed that there is a positive correlation between the primary care physicians (PCPs) beliefs about vaccines and vaccine confidence in the general public [2, 10].

In the previous decade, Poland noted a marked increase of public distrust in vaccinations, reflected by an over 13-fold increase in the number of parents refusing any vaccination of their children, from 3,077 in 2009 to 40,342 in 2018 [11]. The previously mentioned large-scale EU investigation of vaccine confidence highlighted that between 2015 and 2018, Poland noted the largest decrease in public confidence in the importance, effectiveness and safety of vaccines. Previous more detailed investigations [12–16] revealed that Polish residents have a positive overall perception of vaccinations, but poor knowledge of vaccines and vaccine-preventable diseases and concerns about vaccine safety. These investigations also highlighted a major role of physicians as the most used and the most trusted source of information on vaccination for parents.

PCPs are pillars of the Polish childhood immunisation programme, which is coordinated at national level. The immunisation schedule includes a list of seven mandatory vaccines, reimbursed by the state, and seven recommended vaccines, that need to be paid by parents. Vaccinations are carried out in 10,365 vaccination clinics, which are PCP practices that have signed a contract with the national insurer to deliver vaccination services. They regularly receive free of charge vaccine doses from regional stockpiles and report vaccines delivery in relation to each eligible child. The recommended vaccines are available at PCP offices, at the pharmacies and in private healthcare. To meet the legal requirements [17], each vaccination clinic needs to be equipped with anti-shock kit, refrigerator and employ licensed physicians specialised in paediatrics. If there is no paediatrician available, a specialist in family medicine can obtain a license after a vaccinology course or a six-month practice in a vaccination clinic. Only a licensed physician has the right to qualify children for vaccination and prescribe vaccines refunded by the state. The physician is also mandated to inform parents about the possible adverse events following immunisation (AEFI) and inform about recommended vaccines which are not refunded. Following a positive qualification by the physician, a nurse is administering vaccines and completing the documentation. Vaccinations are mandatory by law, which means that parents who refuse are subject to administrative fines. Medical universities in Poland do not offer separate vaccinology modules for students. Medicine and nursing students have vaccinology contents added to relevant courses (ex. Immunology, infectious diseases or paediatrics).

In this investigation, we aimed to assess factors influencing the support for vaccinations among PCPs involved in the childhood immunisation programme, to inform public health policies related to childhood immunisation schedule, as well as education of healthcare workers.

## Material and methods

We collected structured interviews from a representative sample of PCPs working in the childhood immunisation programme across Poland.

### Study design

Between June and July 2017, we carried out a national cross-sectional survey of physicians involved in the qualification and administration of childhood vaccines within the childhood immunisation programme. Because we did not have access to a line list of vaccination clinics, we created a sampling frame of 36,556 PCP practices having at least one specialist in paediatrics and/or family medicine, received from the Chamber of Physicians and Dentists. Subsequently we selected a stratified random sample of practices from this list, with the number of units in each province proportional to the population size of the province. As the next step, we attempted contacting each selected practice to confirm inclusion criteria: (a) Approval of the unit manager for its inclusion; (b) Availability of at least one physician involved in childhood vaccination programme. We included practices until reaching the quota for each province. If necessary, we drew additional samples from the respective provinces sampling frames. We collected a structured telephone interview with one consenting physician in each unit, who was available at the time of the call. The telephone interviews were conducted by a subcontracted marketing research company DSC Research Group.

We calculated the sample size assuming the margin of error to be no more than 5% at the 95% confidence level. We assumed that there were 50,000 physicians specialised in paediatrics or family medicine. Therefore, the minimum required sample was 384 respondents.

### Data collection and variable definitions

The interviews were collected using structured, anonymous computer-assisted telephone interviews (CATI). The questionnaire included 35 items in three parts: (a) sociodemographic and professional information (6 items); (b) knowledge on childhood immunisation including self-assessed knowledge level and six questions on common myths about vaccine safety, impact of vaccines on the infant’s immune system and the immune system capacity to handle multiple vaccines (7 items); (c) sources of information on vaccinations (7 items); (d) adherence to recommendations for physicians working in the childhood immunisation programme (3 items); (e) attitudes towards vaccinations including taking a flu shot every year (5 items); (f) barriers encountered in the administration in the childhood vaccination programme (7 items). The original and translated surveys are included in supportive information (S1 and S2 Files).

We pilot tested the telephone survey with 10 randomly called PCP practices and we subsequently adjusted the wording of some questions, and the instructions for the interviewers.

Since we allowed naming up to three sources of information on immunisation, we categorized them into distinct groups, depending if respondents pointed a single source or more than one. We analysed separately all reported combinations and subsequently, re-categorized them into “scientific” and “non-scientific” sources, considering this distinction as potentially important for building up knowledge and attitudes towards immunisation.

We created an ordinal scale reflecting vaccination support, composed of three equally weighted questions (0–2 points each): a. To which extent does the PCP support childhood vaccinations (No/moderately/strongly); b. If having a child in vaccination age, would the PCP vaccine it? (No/selected vaccines/all recommended vaccines); c. Does the PCP take vaccine against seasonal influenza? (Never/not regularly/every season). The exploratory nature of this investigation and the limited number of questions possible to include in a telephone survey, did not permit us to use previously validated diagnostic scales. We therefore assumed that this ordinal scale will not have diagnostic value, but will reflect the degree to which PCP support the childhood immunisation programme.

Additionally, we created an ordinal scale based on the answers to six common myths about vaccinations, the correct answer to each question contributing 1 point. We identified common myths based on our experience working as editors of the Polish national website www.szczepienia.info, and receiving approximately 1,000 questions annually from the general public. After merging the lowest four categories due to low counts, the final scale ranged from 0 to 3. We also created a variable summarizing adherence to recommendations by PCPs: always informing about the recommended vaccines (on top of mandatory, refunded by the state), and always informing about the possible adverse events following immunisations (AEFI). These two actions are required by law from all physicians working in the childhood immunisation programme.

### Statistical analysis

All statistical analyses were conducted using STATA ver. 15.0 software. First, we described the study population. For categorical variables we calculated frequencies and proportions. For ordinal variables we calculated medians and interquartile ranges. For interval/ratio variables, we calculated means and standard deviations. We calculated the correlation between ordinal score variables using Spearman correlation coefficients.

We used the ordered logistic regression to investigate factors independently influencing PCP support for vaccinations. We reported crude and adjusted proportional odds ratios and 95% confidence intervals for one unit increase in the score reflecting vaccination support level, given that the other variables in the model are held constant.

To describe the subgroup of PCP least supporting vaccinations, we used frequencies and proportions. To compare the distribution of categorical variables we used chi-square test and reported Pearson p-values. We assumed a significance level of 5% for reporting results.

### Ethical considerations

Because the survey did not concern patients, was anonymous, and did not contain any sensitive information, the Institutional Review Board of the National Institute of Public Health—National Institute of Hygiene applied an exemption from the full ethical review. The respondents did not receive any incentive to participate. Before the start of the interview, each respondent was informed about the purpose of the study, its confidentiality and was asked for oral consent. If no consent was given, the interview was terminated. The data were collected and analysed anonymously. We assured safe storage of interview recordings for quality assurance purposes.

## Results

**Fig 1:**
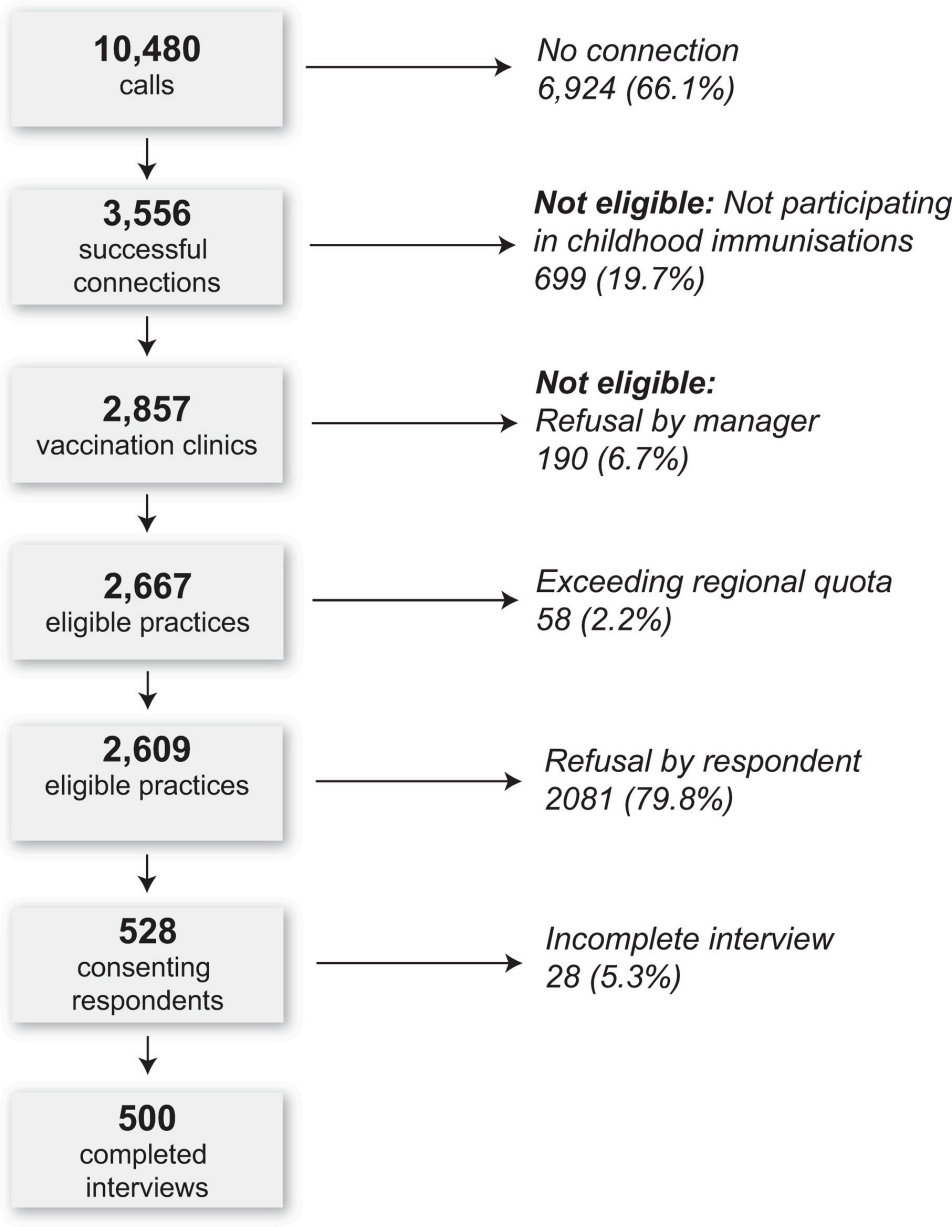
Recruitment of the study sites and respondents within the units, Poland, June-July, 2017.

Of the 2,609 eligible respondents reached, 500 were successfully interviewed, leading to a response rate of 19% (Fig 1). Among the most common reasons for refusal noted by the interviewers were: lack of time, distrust of surveys and polls, sensitive topic and concern about potential consequences of participation.

### Study participants

Of the 500 respondents, 377 (75.4%) were female (Table 1). The mean age was 55 years (median: 56 years). Accordingly, the majority had professional experience exceeding 25 years. The localization of general practices across provinces reflected well the proportional distribution of the Polish population. 403 respondents (81%) were paediatricians, and the remaining 97 (19%) were family doctors.

**Table 1:** Descriptive characteristics of the studied population, Poland, June-July 2017.

Characteristic	Categories	n (%)
Sex	Male	123 (24.6%)
	Female	377 (75.4%)
Age (years)	<40	46 (9.2%)
	40–49	89 (17.8%)
	50–59	171 (34.2%)
	>60	194 (38.8%)
Medical specialty	Paediatrician	403 (80.6%)
	Other	97 (19.4%)
Province	Dolnośląskie	35 (7%)
	Kujawsko-pomorskie	26 (5.2%)
	Lubelskie	28 (5.6%)
	Lubuskie	13 (2.6%)
	Łódzkie	30 (6%)
	Małopolskie	48 (9.6%)
	Mazowieckie	76 (15.2%)
	Opolskie	12 (2.4%)
	Podkarpackie	27 (5.4%)
	Podlaskie	15 (3%)
	Pomorskie	33 (6.6%)
	Śląskie	56 (11.2%)
	Świętokrzyskie	14 (2.8%)
	Warmińsko-mazurskie	18 (3.6%)
	Wielkopolskie	49 (9.8%)
	Zachodniopomorskie	20 (4%)
Self-assessed knowledge:	Poor	2 (0.4%)
	Median	34 (6.8%)
	Good	270 (54.4%)
	Very good	190 (38.3%)
Using two sources of information	Training courses & scientific literature	182 (36.4%)
	Scientific literature & websites	56 (11.2%)
	Training courses & websites	28 (5.6%)
	Scientific literature & official documents	22 (4.4%)
	Training courses & official documents	11 (2.2%)
	Websites & official documents	11 (2.2%)
	Scientific literature & pharmaceutical representatives	11 (2.2%)
	Training courses & pharmaceutical representatives	11 (2.2%)
	Other physicians & scientific literature	7 (1.4%)
	Scientific literature & social media	6 (1.2%)
	Websites & pharmaceutical representatives	4 (0.8%)
	Other combinations of sources	6 (1.2%)
Using one source of information	Scientific literature	72 (14.4%)
	Training courses	32 (6.4%)
	Official documents	20 (4.0%)
	Websites	14 (2.8%)
	Pharmaceutical representatives	4 (0.8%)
	Other sources	3 (0.6%)
Attended a conference/workshop in preceding year		316 (63.2%)
Offers recommended vaccines to every patient		492 (98.4%)
Always informs about adverse events following immunisation		467 (93.4%)
**Correct (negative) answers to common myths about vaccinations**;		
Vaccines can cause autism		463 (92.6%)
MMR vaccine contains thimerosal		434 (86.8%)
Too many antigens weaken the child immunisation system		468 (93.6%)
Vaccines are produced from human tissues and organs		438 (87.6%)
Vaccine adverse events are more dangerous than the disease itself		475 (95.0%)
The vaccine prevents 100% cases of the disease		372 (74.4%)

The most commonly used sources of information on vaccinations were scientific literature and manuals (72%), scientific training courses (53%), internet resources (websites and social media, 23%) and official documents from Public Health institutions (13%). 355 respondents (71%) reported more than one information source. Among the combinations of sources, the most common were the use of both scientific sources (training courses with literature, 51%), literature with internet resources (16%) and training courses with internet resources (8%) (Table 1). 145 (29%) physicians reported a single information source, of which 50% used scientific literature, 22% training courses, 14% official documents, and 10% internet resources.

Four-hundred and sixty-one (92.2%) physicians followed recommendations of the childhood immunisation programme by offering recommended vaccinations and informing about AEFIs. The average self-assessed knowledge (range 0–5) was 4.31 (SD 0.61). The score based on addressing anti-vaccination myths (range 0–3) was 2.34 (SD 0.82). There was no correlation between the self-assessed knowledge and addressing anti-vaccination myths (r_s_ = -0.0006, p = 0.9891).

### Physicians support for vaccinations

**Fig 2:**
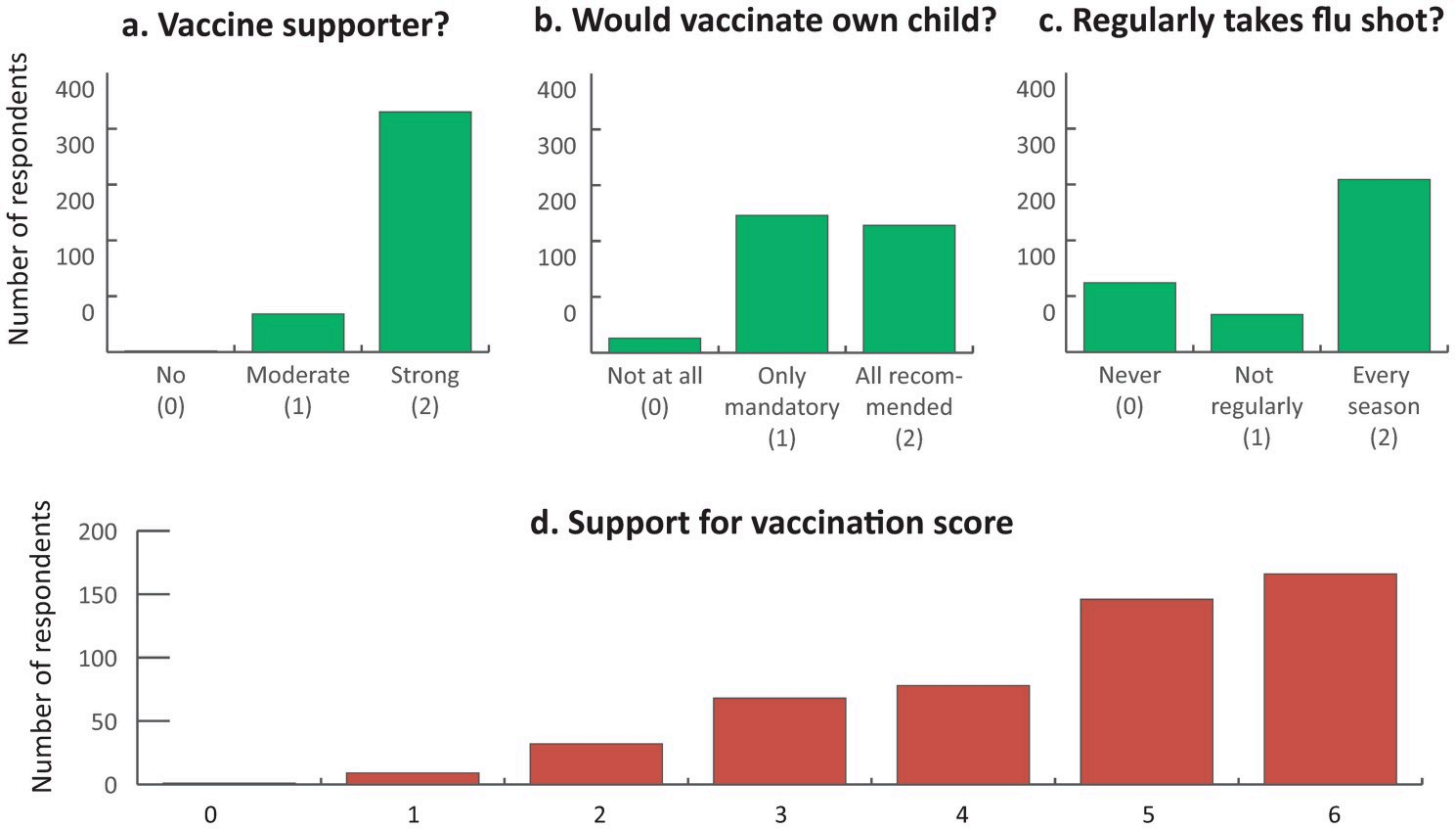
Support for vaccinations scale construction, based on answers to three questionnaire items graded 0–2.

Fig 2 summarizes answers to the three questions concerning physician’s attitudes used to calculate the scale reflecting PCP support for vaccinations. Eighty-six percent of respondents described themselves as strong supporters of immunisations. Forty-six percent of respondents declared that if confronted with the decision to vaccinate of their own children, they would decide to give them all mandatory (reimbursed by the state) and recommended (paid) vaccinations. Sixty-two percent of respondents took each year the recommended vaccine against seasonal influenza. The median vaccination support score (0–6) among study participants was 5 (IQR 2).

### Association of explanatory variables on PCP support for vaccinations

There was no significant effect of sex on vaccination support (Table 2). The effect of paediatric specialisation, self-perceived knowledge, attending a conference or workshop on vaccinations in preceding year and adherence to recommendations, seen in the univariable analysis, was no longer significant, when taking into account other variables in the multivariable analysis. We found a strong effect of increasing age on support for vaccinations. The association was not significant in the univariable analysis, but found to be significant after adjusting to other variables in the multivariable analysis. Working in two provinces, one located in the north-west (Zachodniopomorskie) and one in the south-east of Poland (Malopolskie) had negative effect on vaccination support, but only the former remained significant in the final model. The remaining variables—addressing vaccination myths, and use of different sources of information, were significantly associated with support for vaccinations. Regarding the sources of information, using scientific sources had a strong and significant effect both among respondents using two sources and a single source of information (Table 2).

**Table 2:** Univariable and multivariable associations between respondent characteristics and vaccination support, Poland, June-July, 2017.

Variables	OR	SE	p	95% CI	aOR	SE	p	95%CI
Sex (male vs. female)	0.74	0.14	0.107	0.52–1.07	1.03	0.21	0.885	0.69–1.54
Age group (ref <40 years)								
40–49 years	1.12	0.35	0.718	0.61–2.07	1.03	0.34	0.923	0.54–1.97
50–59 years	1.65	0.47	0.080	0.94–2.90	1.72	0.53	0.079	0.94–3.17
60+ years	1.74	0.49	0.052	0.99–3.03	**1.97**	**0.60**	**0.025**	**1.09–3.58**
Province (Ref: Dolnośląskie)								
Kujawsko-pomorskie	0.58	0.28	0.264	0.22–1.51	0.66	0.33	0.406	0.25–1.74
Lubelskie	0.79	0.36	0.613	0.33–1.93	0.86	0.40	0.476	0.34–2.15
Lubuskie	0.79	0.48	0.698	0.24–2.59	0.72	0.44	0.592	0.22–2.36
Łódzkie	1.02	0.46	0.967	0.42–2.45	1.12	0.53	0.815	0.44–2.85
Małopolskie	**0.37**	**0.15**	**0.013**	**0.17–0.81**	0.48	0.20	0.075	0.21–1.08
Mazowieckie	0.62	0.23	0.200	0.30–1.29	0.89	0.35	0.778	0.41–1.94
Opolskie	1.63	1.08	0.460	0.45–5.95	1.74	1.18	0.414	0.46–6.57
Podkarpackie	0.96	0.46	0.933	0.38–2.45	1.17	0.56	0.744	0.46–3.00
Podlaskie	0.88	0.52	0.827	0.28–2.78	1.04	0.61	0.950	0.33–3.28
Pomorskie	0.72	0.33	0.476	0.30–1.75	0.98	0.46	0.974	0.40–2.44
Śląskie	0.62	0.25	0.236	0.29–1.36	0.61	0.25	0.231	0.27–1.37
Świętokrzyskie	0.66	0.35	0.536	0.23–1.89	0.51	0.28	0.227	0.18–1.51
Warmińsko-mazurskie	0.56	0.29	0.255	0.21–1.52	0.67	0.35	0.453	0.24–1.88
Wielkopolskie	0.89	0.36	0.778	0.40–1.97	0.94	0.39	0.889	0.42–2.14
Zachodniopomorskie	**0.17**	**0.08**	**<0.001**	**0.07–0.45**	**0.21**	**0.11**	**0.003**	**0.08–0.59**
Paediatrician vs other specialty	**1.63**	**0.33**	**0.016**	**1.09–2.42**	1.09	2.25	0.690	0.70–1.70
Self-perceived knowledge (Ref: Less than good)								
Good	1.19	0.35	0.550	0.67–2.11	1.02	0.32	0.945	0.55–1.89
Very good	**2.05**	**0.62**	**0.018**	**1.13–3.70**	1.51	0.50	0.214	0.79–2.91
Addressing vaccination myths	**1.48**	**0.15**	**<0.001**	**1.22–1.80**	**1.57**	**0.17**	**<0.001**	**1.27–1.94**
Adherence to recommendations	**1.84**	**0.55**	**0.039**	**1.03–3.30**	1.76	0.55	0.072	0.95–3.26
Attended a training in preceding year	**1.48**	**0.25**	**0.018**	**1.07–2.05**	1.06	0.20	0.757	0.73–1.54
Sources of information (Ref: Two sources. both non-scientific)								
Two sources, of which one scientific	2.06	0.84	0.078	0.92–4.60	1.90	0.83	0.139	0.81–4.47
Two sources, both scientific	**3.53**	**1.45**	**0.002**	**1.58–7.88**	**2.68**	**1.19**	**0.026**	**1.13–6.40**
One source, non-scientific	**2.78**	**1.32**	**0.032**	**1.09–7.07**	2.29	1.14	0.094	0.87–6.07
One source, scientific	**3.38**	**1.44**	**0.004**	**1.47–7.79**	**3.09**	**1.39**	**0.012**	**1.29–7.45**

### Characterisation of PCPs who are least supportive for vaccinations

Among 42 physicians whose support for vaccination was lowest (score < = 2), only eight considered themselves strong vaccination supporters, none would vaccinate their children with all recommended vaccines on top of the mandatory ones, and none took the flu shot every year. Compared with remaining respondents, the least supportive PCPs had lower median age (51 vs. 56 years), were more frequently men (29% vs. 24%), and attended a workshop or conference on vaccinations less commonly in the previous year (50% vs. 64%, p = 0.064). Compared with remaining respondents, the least supportive PCPs were using more frequently information from scientific literature (76% vs. 71%, p = 0.491), but less frequently from training courses (31% vs. 55%, p = 0.003).

Compared with remaining respondents, the least supportive PCPs did more frequently agree with vaccination myths, especially those related with vaccine safety. Eight of them agreed that vaccines can cause autism (19% vs. 6%, p = 0.003). Fifteen of the least supportive respondents agreed that MMR vaccine contains thimerosal (36% vs. 11%, p<0.001). Twelve of the least supportive respondents agreed that many antigens in combined vaccines are weakening the child’s immune system (29% vs. 4%, p<0.001). Five of the least supportive respondents agreed that AEFIs are more dangerous than the disease against which vaccines protect (12% vs. 4%, p = 0.032). Addressing of the remaining myths was not significantly different between the two groups.

Compared with remaining respondents, the least supportive PCPs agreed more frequently that the barrier for informing parents about vaccinations was lack of arguments to address parents’ concerns (19% vs. 12%, p = 0.172). The least supportive PCPs identified less frequently as a barrier their own communication skills (2% vs. 9%, p = 0.125). None of the differences in the barriers for informing parents about vaccinations were statistically significant.

## Discussion

We investigated the sources of information and support for vaccinations among PCPs in the period of increasing vaccine skepticism in Poland. Support for vaccinations was associated with increasing age, exclusive use of scientific sources of information, and addressing vaccination myths. We also observed regional variations in PCP vaccination support. Even if we did not aim to study vaccine hesitancy among PCP, we identified a near 10% subgroup of vaccinating physicians who seemed to have doubts about the safety of vaccines. Because it was an exploratory, hypothesis-generating investigation, below we tried to identify areas for future research of vaccine confidence in Poland.

We found significant associations between increasing age and region of practice on PCP support for vaccinations. The effect of age may reflect the lower practical experience of younger physicians who see less infectious disease cases, compared to older colleagues. Surveys in UK and Italy had found that younger, more recently qualified physicians feel less confident and knowledgeable about vaccines and have more problems in addressing parents’ questions and concerns, compared to older colleagues [18, 19]. Furthermore, a survey among PCPs in France showed that they recommended vaccines more frequently when they felt comfortable explaining vaccines benefits and risks to patients or trusted official sources of information [20]. The lower support for vaccinations among younger PCPs can also indicate the influence of anti-vaccination messages in social media. We also found regional differences in PCP support for vaccination, which may be related to differences in post-graduate education and communication on vaccination by the regional public health services. To investigate the regional determinants of vaccine confidence, Poland could follow the example of Italy which is systematically investigated determinants of vaccine hesitancy in parallel national representative samples of the general public, parents and healthcare professionals [18, 21–24].

Exclusive use of scientific sources of information and addressing vaccination myths were most strongly associated with vaccination support. These associations could result both from the positive role of knowledge of vaccines and immunisation, and from the possible ambiguous influence of non-scientific sources of information, like opinions of colleagues, opinions of patients, websites and/or social media. The association between knowledge and vaccine confidence among healthcare workers (HCWs) has been documented previously [25–27]. Physicians working in vaccination clinics should in theory have up to date knowledge on particular vaccines and vaccine-preventable diseases. The legal requirements for vaccinators include obtaining a medical specialty in paediatrics or being a family doctor with at least 6-month practical experience in administering vaccines, and taking a vaccinology course [17]. However, once the physician obtains the license to administer childhood vaccines, there is no formal requirement of continuous education. There is only a requirement to accumulate a minimum number of CME points every 5 years to keep the medical specialty. Thus physicians decide individually whether to use evidence-based materials in their practice and participate in certified vaccinology courses. In our investigation, attending a vaccination module in the preceding year was not significantly associated with PCP vaccination support. However, we found out that PCPs least supportive for vaccinations were less frequently attending vaccinology courses, compared to the remaining physicians. Future research in Poland should therefore address improved accessibility of evidence-based information on vaccines and vaccine-preventable diseases, including both the appropriateness of contents and of communication channels for different healthcare groups.

In 2018, an EU-wide study of PCP vaccine confidence involved 100 practitioners in Poland [2]. The authors found a high overall vaccine confidence among Polish PCPs in the importance, safety, effectiveness of vaccines, not corresponding with decreasing vaccination confidence in the general public. However, Polish PCPs demonstrated highest religious compatibility concerns among all studied countries (29% vs. 12% average in 10 studied countries). It could be a reflection of the perceptions in the general public, noted in a parallel study among 1022 randomly selected respondents. In this survey, 41% representatives of the general public expressed concerns that vaccines were not compatible with their religious beliefs, a figure considerably higher than the EU average of 22%. Based on the above considerations, future vaccine confidence research should address specific determinants of vaccine confidence, including moral and religious beliefs.

We found a signal of low vaccine confidence among a fraction of Polish PCPs. Although the proportion of practitioners least supporting vaccinations was not very high, this is worrying since we focused on the “vaccinology elite”, the best trained professionals who are entrusted with the implementation of the childhood immunisation programme. As highlighted in a recent investigation of Karlsson et al., HCWs confidence in the benefits and safety of vaccines is increasing along with the degree of medical training [28]. Thus, our result may signal a more serious problem among HCWs in Poland. We found that the least supportive PCPs expressed highest concerns about the safety of childhood vaccinations. Previous investigations have linked decreased vaccine confidence with concerns about vaccine safety [9, 20, 25, 28, 29]. Furthermore, only 46% PCPs in our sample declared the (potential) willingness to vaccinate their own children using all mandatory and recommended vaccines. This decision may reflect both the high cost and the perception of lesser importance of recommended vaccines. Divergent attitudes of HCWs regarding immunisation of their relatives and their patients, may reflect the need for autonomy in making personal decisions [30, 31]. Although it is possible that the PCPs decision to not vaccinate their own children will not affect their recommendation to vaccinate their patients, it is indirectly reflecting their overall vaccine confidence. Future research is necessary to understand the determinants of low confidence in both vaccinating physicians and all HCWs.

The problem of vaccine hesitancy among HCWs is increasingly recognized globally. Paterson et al. identified 185 studies on vaccine hesitancy among HCWs from 33 countries [25]. This review pointed out that knowledge about particular vaccines helped building HCWs own vaccine confidence and their willingness to recommend vaccinations to others. According to the review, being vaccinated itself or being trained to give advice, contributed to a better vaccine acceptance among HCWs. To address the problem of vaccine hesitancy, several authors have emphasized designing and evaluating communication tools aimed at different professional groups [20], as well as strengthening trust between healthcare practitioners and the health authorities [32]. All above mentioned authors stress the need to improve HCW education and increase accessibility of reliable evidence-based information on vaccines and vaccine-preventable diseases. Considering the complexity of the vaccine confidence determinants, future vaccine confidence research should not only focus on vaccinating PCPs, but also on other healthcare professionals, including hospital physicians, nurses, pharmacists, but also address media influencers and politicians.

Our investigation had several limitations. First, the low response ratio could affect the external representativeness of our study population. This would happen if the non-respondents would systematically differ from respondents in terms of support for vaccinations. Indeed, the more busy and younger physicians might have not responded to our telephone calls. On the other hand, the demographic structure of our study participants reflect well the population of physicians with specialisation in paediatrics, whose average age in 2017 was 55.8 years (median 56 years) [33]. We tried to address this potential limitation in the design phase by planning telephone calls at different hours of day and their flexible scheduling, and in the analysis phase by adjusting the multivariable analysis for age. Second, there could be potential information bias related to very short interviews (average duration 9.5 minutes), which were dictated by the telephone conversation restrains and very busy respondents. This means that we had to rely on spontaneous answers without room for clarifications or corrections. This could lead to some imprecision and mistakes in the collected interviews, both from the side of the respondent and the interviewer. We addressed the possible information bias by thorough two-stage quality control, first by the supervisor during the interviews, second by validation 10% recorded interviews by the study coordinator. Third, the questions constituting the vaccination support scale are related to each other and with some explanatory variables, which could influence the multivariable associations. For example, vaccination against seasonal influenza is associated with age, since both age over 55 years and being a healthcare worker constitutes an indication for seasonal influenza vaccination. We cannot disentangle to which extent taking the vaccine each year was related to the respondent’s attitude towards vaccinations or to his/her medical indications for vaccination.

Use of valid psychometric scales needs appropriate conditions and a short telephone conversation is not an appropriate setting for long, carefully controlled interviews. Therefore, our investigation had an exploratory, and hypothesis-generating nature. Hopefully, it will trigger new investigations and evidence based interventions to countermeasure the increasing problem of vaccine hesitancy in Poland.

## Conclusions

Increasing age, exclusive use of scientific sources and addressing vaccination myths, were most strongly associated with PCP support for vaccinations. Since we investigated only one subgroup of healthcare professionals, presumably most informed on vaccines and immunisations, more in-depth investigations should be continued, addressing different healthcare professionals. Future research should employ carefully designed and validated psychometric tools and address not only knowledge and basic determinants of vaccine confidence, but also study moral and religious determinants of attitudes towards vaccinations among different stakeholders.

Based on our exploratory investigation, we recommend the improvement of the amount and quality of evidence-based educational materials that should be easily available to PCPs. As a long term measure, we recommend the revision of graduate and post-graduate curricula of healthcare workers training to include more vaccinology contents.

## Supporting information

S1 File Full original survey in Polish. (DOCX)

S2 File Survey translated in English. (DOCX)
