# Supplementary material for: Exploring factors improving support for vaccinations among Polish primary care physicians
Source: PLoS One. 2020 May 1;15(5):e0232722. doi: 10.1371/journal.pone.0232722 (PMC7194393; doi:10.1371/journal.pone.0232722)
Supplement: S1 File — (DOCX) [file pone.0232722.s001.docx]

Supplementary file 1. Original survey of knowledge and attitudes of physicians towards vaccinations, Poland, July 2017.

**Tytuł badania: Ocena wiedzy i postaw lekarzy szczepiących dzieci na temat szczepień**

Pytania wprowadzające:

**I. Płeć**

1. Kobieta
2. Mężczyzna

**II. Ile ma pani/Pan lat?**

………......

**III. Jaki jest Pani/Pana staż pracy?**

….............

**IV. Czy ma Pani/Pan dzieci?**

1. Kobieta
2. Mężczyzna
3. **Proszę określić swój stan wiedzy na temat szczepień**

*(w skali od 1 do 5, gdzie 1 oznacza „bardzo zły”, a 5 oznacza „bardzo dobry”)*

1 2 3 4 5

1. **Skąd czerpie Pan/Pani swoją wiedzę na temat aktualnych szczepień?**

*(proszę o wskazanie dwóch głównych źródeł)*

*[pytanie prekategoryzowane; kategorie odpowiedzi dla ankietera: Internet, szkolenia, inni lekarze, znajomi/rodzina, literatura fachowa/podręczniki, media/media społecznościowe]*

1. **Czy w 2016 roku uczestniczył Pan/uczestniczyła Pani w szkoleniu/konferencji/warsztacie dotyczącym szczepień?**

a. tak

b. nie

c. *nie pamiętam* (nie czytamy)

1. **Czy o kalendarzu szczepień z rodzicami dziecka rozmawia Pan/Pani:**

a. podczas rutynowej wizyty kontrolnej

b. w trakcie specjalnej wizyty rodziców w gabinecie poświęconej tylko szczepieniom

c. w ogóle nie rozmawiam

1. **Załóżmy, że musi Pan/Pani zdecydować o zaszczepieniu swojego, nowonarodzonego dziecka, u którego nie ma żadnych przeciwskazań do szczepień. Co Pan/Pani robi?**

a. realizuję tylko szczepienia obowiązkowe

b. realizuję szczepienia obowiązkowe i wybrane szczepienia zalecane

c. realizuję szczepienia obowiązkowe i wszystkie szczepienia zalecane

d. nie szczepię swojego dziecka w ogóle

1. **Czy informuje Pan/Pani rodziców dziecka o szczepieniach zalecanych?**

a. tak, zawsze

b. czasami

c. nie, nigdy, nie mam takiego zwyczaju

1. **Czy podczas rozmowy o szczepieniach informuje Pan/Pani rodziców dziecka o możliwości wystąpienia niepożądanych odczynów poszczepiennych (NOP)?**

a. tak, zawsze

b. czasami

c. nie, nigdy, nie mam takiego zwyczaju

1. **Jak często spotyka się Pan/Pani z odmową szczepień ze strony rodziców?**

a. kilka razy w miesiącu

b. kilka razy w roku

c. raz w roku

d. raz na kilka lat

e. nigdy się nie spotkałem/nie spotkałam

1. **Co jest dla Pana/Pani największym problemem, jeśli chodzi o rozmowę z rodzicami dziecka na temat szczepień?**

*(odpowiedzi proszę zaznaczyć na skali od 1 do 5, gdzie 1 oznacza „zdecydowanie się nie zgadzam”, a 5 oznacza „zdecydowanie się zgadzam”)*

a. brak mojej aktualnej wiedzy na temat szczepionek

1 2 3 4 5

b. brak umiejętności komunikacji z rodzicami dzieci

1 2 3 4 5

c. brak argumentów na przedstawiane obawy rodziców

1 2 3 4 5

d. brak materiałów edukacyjnych w sieci, do których mogę odesłać rodziców

1 2 3 4 5

e. negatywne nastawienie rodziców do szczepień

1 2 3 4 5

f. brak czasu

1 2 3 4 5

1. **Z którą z poniższych postaw identyfikuje się Pan/Pani najbardziej?**

a. zdecydowany zwolennik/zdecydowana zwolenniczka szczepień

b. umiarkowany zwolennik/umiarkowana zwolenniczka szczepień

c. umiarkowany przeciwnik/umiarkowana przeciwniczka szczepień

d. zdecydowany przeciwnik/zdecydowana przeciwniczka szczepień

e. nie jestem ani zwolennikiem/zwolenniczką, ani przeciwnikiem/przeciwniczką szczepień

1. **Przeczytam Panu/Pani kilka opinii na temat szczepień. Proszę powiedzieć, z którymi się Pan/Pani zgadza lub nie zgadza.**

a. Szczepionki mogą wywoływać autyzm.

zgadzam się/nie zgadzam się

b. Szczepionka przeciwko MMR zawiera szkodliwy dla zdrowia dziecka tiomersal.

zgadzam się/nie zgadzam się

c. Podając wiele antygenów w szczepionce skojarzonej osłabia się układ immunologiczny dziecka.

zgadzam się/nie zgadzam się

d. Szczepionki pochodzą z materiału pobranego z narządów człowieka.

zgadzam się/nie zgadzam się

e. Niepożądany odczyn poszczepienny jest zazwyczaj bardziej niebezpieczny niż objaw choroby.

zgadzam się/nie zgadzam się

f. Szczepionka zapewnia całkowitą ochronę przed zachorowaniem na daną chorobę

zgadzam się/nie zgadzam się

1. **Czy szczepi się Pan/Pani przeciwko grypie?**

a. tak, regularnie co roku lub niemalże co roku

b. nieregularnie, od czasu do czasu

c. nie, nigdy się nie szczepię *(proszę o przejście do pytania 13.)*

1. **Nie szczepi się Pan/Pani przeciwko grypie, ponieważ:**

a. uważam, że ta szczepionka przeciwko grypie jest nieskuteczna

b. po zaszczepieniu zachorowałem/chorowałam

c. boję się wystąpienia NOP-u

d. inne
