# Supplementary material for: Exploring factors improving support for vaccinations among Polish primary care physicians
Source: PLoS One. 2020 May 1;15(5):e0232722. doi: 10.1371/journal.pone.0232722 (PMC7194393; doi:10.1371/journal.pone.0232722)
Supplement: S2 File — (DOCX) [file pone.0232722.s002.docx]

Supplementary file 2. Translated survey of knowledge and attitudes of physicians towards vaccinations, Poland, July 2017.

Introductory questions:

**I. Respondent sex**

1. Female
2. Male

**II. Respondent age**

________

**III. How long are you working as physician?**

________

**IV. Do you have children?**

1. Female
2. Male
3. **Please indicate how do you perceive your knowledge on vaccinations**

*(in a scale from 1 to 5, where 1 means „very basic” and 5 means „very advanced”)*

1 2 3 4 5

1. **Where do you find current information on vaccinations?**

*(please indicate your two main sources subsequently categorized by the interviewer using pre-defined categories: Internet, training, other physicians, family/friends, scientific publications, manuals, media, social media)*

1. **Did you participate in a training/conference/workshop concerning vaccinations in 2006?**

a. Yes

b. No

c. Don’t remember (do not read out)

1. **Do you talk with the child’s parents about the immunization schedule:**

a. during a routine healthy child’s visit?

b. during a scheduled visit only dedicated to vaccinations?

c. don’t talk about this at all?

1. **Let’s imagine, that you have to decide whether to vaccinate your new born child, when there is no contraindications for vaccinations. What would you do?**

a. I would only apply the mandatory vaccinations included in the immunization schedule

b. I would apply the mandatory and selected recommended vaccinations

c. I would apply the mandatory and all recommended vaccinations

d. I would not vaccinate my child at all.

1. **Do you inform the child’s parents about recommended vaccinations?**

a. Yes, always

b. Sometimes

c. No, never

1. **Do you inform the child’s parents about the possibility of adverse events following immunisation, when you talk about vaccinations?**

a. Yes, always

b. Sometimes

c. No, never

1. **How often are you facing refusal of parents to vaccinate their child?**

a. Several times per month

b. Several times per year

c. Once per year

d. Once per few years

e. Did not experience such issue

1. **What is the biggest problem for you during the talks with parents about vaccinations?**

*(please indicate your answer in a scale from 1 to 5, where 1 means „strongly disagree”, and 5 means „strongly agree”)*

a. Lack of my up-to-date knowledge on vaccinations

1 2 3 4 5

b. Lack of communication skills with the child’s parents

1 2 3 4 5

c. Lack of arguments to address the raised parents’ concerns

1 2 3 4 5

d. Lack of educational materials in the Internet, to which I can refer the parents

1 2 3 4 5

e. Negative parent’s attitudes towards vaccinations

1 2 3 4 5

f. Lack of time

1 2 3 4 5

1. **Which attitude do you identify with most?**

a. Determined supporter of vaccinations

b. Moderate supporter of vaccinations

c. Moderate opponent of vaccinations

d. Determined opponent of vaccinations

e. Neither supporter nor opponent of vaccinations

1. **I will read some opinions on vaccinations. Please indicate with which you agree and with which - not**

a. Vaccines can cause autism.

Agree / Disagree

b. The MMR vaccine contains thimerosal which is harmful for the child’s health

Agree / Disagree

c. By giving many antigens in combined vaccines we are weakening the child’s immunization system.

Agree / Disagree

d. Vaccines are produced from materials taken from human organs.

Agree / Disagree

e. Adverse events following immunization are usually more dangerous than the disease symptoms.

Agree / Disagree

f. The vaccine completely prevents the development of the disease,

Agree / Disagree

1. **Do you vaccinate yourself against seasonal influenza?**

a. Yes, I take the vaccine every year or almost every year

b. Yes, but not regularly, every few years

c. No, I never take the vaccine *(please go to question 13.)*

1. **What are the reasons to not vaccinate yourself against seasonal flu?**

a. I think that the seasonal flu vaccine is not effective

b. I was sick after one earlier vaccination

c. I have concerns about the adverse events following immunization

d. Other
